# *In Situ* Measurement of the Junction Temperature of Light Emitting Diodes Using a Flexible Micro Temperature Sensor

**DOI:** 10.3390/s90705068

**Published:** 2009-06-26

**Authors:** Chi-Yuan Lee, Ay Su, Yin-Chieh Liu, Wei-Yuan Fan, Wei-Jung Hsieh

**Affiliations:** Department of Mechanical Engineering, Yuan Ze Fuel Cell Center, Yuan Ze University, Taoyuan, Taiwan; E-Mails: meaysu@saturn.yzu.edu.tw (A.S.); M77YCLIU@saturn.yzu.edu.tw (Y.-C.L.); s975040@mail.yzu.edu.tw (W.-Y.F.); s965034@mail.yzu.edu.tw (W.-J.H.)

**Keywords:** LED, junction temperature, MEMS, flexible micro temperature sensor

## Abstract

This investigation aimed to fabricate a flexible micro resistive temperature sensor to measure the junction temperature of a light emitting diode (LED). The junction temperature is typically measured using a thermal resistance measurement approach. This approach is limited in that no standard regulates the timing of data capture. This work presents a micro temperature sensor that can measure temperature stably and continuously, and has the advantages of being lightweight and able to monitor junction temperatures in real time. Micro-electro-mechanical-systems (MEMS) technologies are employed to minimize the size of a temperature sensor that is constructed on a stainless steel foil substrate (SS-304 with 30 μm thickness). A flexible micro resistive temperature sensor can be fixed between the LED chip and the frame. The junction temperature of the LED can be measured from the linear relationship between the temperature and the resistance. The sensitivity of the micro temperature sensor is 0.059 ± 0.004 Ω/°C. The temperature of the commercial CREE^®^ EZ1000 chip is 119.97 °C when it is thermally stable, as measured using the micro temperature sensor; however, it was 126.9 °C, when measured by thermal resistance measurement. The micro temperature sensor can be used to replace thermal resistance measurement and performs reliably.

## 1. Introduction

A light emitting diode (LED) is fabricated from p-type and n-type semiconductor materials, and an input voltage causes the LED chip to glow by combining electron holes and electrons at the p-n junction. An LED emits various colors, which are determined by the combined semiconductors. The advantages of an LED over a light-bulb include its small volume, low temperature, low power consumption, long lifetime, fast response and environmental friendliness, whereas the standard light-bulb is limited in terms of high power consumption, ease of breakage and mercury pollution. LEDs are expected to replace all conventional light-bulbs in the next decade. Eight percent of the input power of an LED is converted to thermal energy; the area of epitaxy is very small, and the heat flux per unit area exceeds that of a central processing unit (CPU). LEDs with a high heat flux output require a strongly conducting radiator, to prevent the destruction of the package of the epitaxy [1].

The temperature of the junction affects LED performance in several ways. The light output center wavelength, spectrum, power and diode reliability all depend directly on the junction temperature, which in an LED cannot be measured using currently available instruments. Accordingly, Siegal [2] utilized the principle of a diode to indirectly measure the junction temperature in an LED. Although some investigations have determined junction temperatures by estimating thermal resistance, such an indirect method is inaccurate [3,4]. Kim [5] used thermal resistance measurement to study the high-power GaN-based light emitting diode with multichip design, and exploited the thermal design rule for packaging high-power multichip LEDs. Chen [6] measured the junction temperature using this approach, and obtained the results that were consistent with those obtained using the emission peak energy shift approach. The infrared ray scheme is an effective means of measuring the temperature of an LED, but it measures only the temperature on the top of the chip, which is not the junction temperature. Senawiratne [7] estimated used thermal resistance measurement to determine the measurement junction temperature of a light emitting diode, increasing the driving currents in increments at increments of 10 mA to 250 mA. Xi [8] examined a theoretical model of the dependence of the forward voltage of a diode on junction temperature. Chen [9] applied micro-electro-mechanical systems (MEMS) techniques to develop a resistance temperature detector (RTD). Chen [10] also adopted Y-doped BaZrO_3_ thin films to make an RTD high-temperature sensor, and the resistance temperature detector is also applied in numerous domains [11,12]; this method is quite effective. Although a thermal resistance measurement is frequently employed, it is inaccurate because a change in the current produces a pulse, but the current can only be measured when it is stable.

This work presents a novel approach based on micro-electro-mechanical-systems (MEMS) technology to fabricate a flexible micro temperature sensor on a stainless steel foil substrate (SS-304 with a thickness of 30 μm) in order to monitor *in-situ* the junction temperature of a light emitting diode. This fabrication technique has the advantages of: (1) small size, (2) high sensitivity, (3) flexible but precise measurement positions, and (4) mass production.

## 2. Methodology

### 2.1. Thermal Resistance Measurement

**Figure 1.**
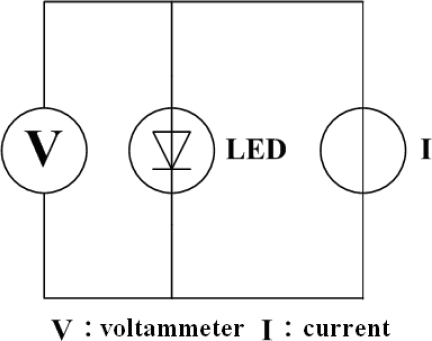
Circuit illustration of thermal resistance measurement technology.

**Figure 2.**
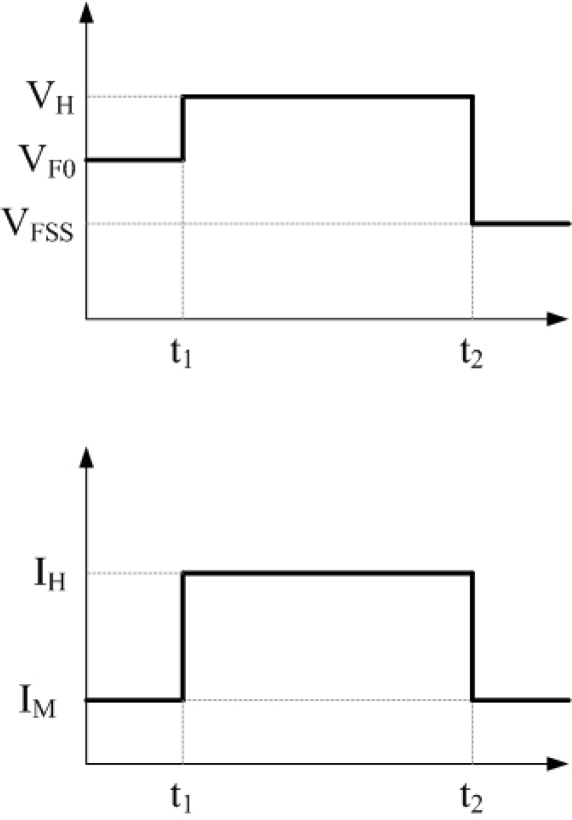
Measuring procedure of thermal resistance measurement technology.

Previously, the junction temperature of LEDs has been determined from changes in the forward voltage [13]. Thermal resistance was measured by exploiting the fact that resistance increases with temperature, and the voltage thus declines. Figure 1 presents the circuit diagram. Thermal resistance is measured as follows: (1) Input an initial measured current (I_M_) and measure the initial voltage (V_F0_); (2) Input a higher heating current (I_H_) until the temperature is stable; (3) Change the measured current (I_M_) rapidly, and measure the voltage (V_FSS_) after heating; (4) Compare V_F0_ and V_FSS_ following calibration to yield the temperature of the LED chip, as shown in Figure 2.

Although measurements of thermal resistance are useful, this approach cannot always be applied to measure temperature, and a pulse is produced when the current is changed. The pulse affects the voltage, destabilizing the measured temperature.

### 2.2. Resistance Temperature Detector

In this work, the temperature sensor was a resistance temperature detector (RTD). As the environmental temperature increases, the resistance of the RTD also increases, because a metal conductor has a positive temperature coefficient (PTC). When the temperature of the RTD varies linearly, the relationship between the measured resistance and the change in temperature can be expressed as:

(1) $$R_{t}=R_{i}\;\left(1+\alpha_{T}\;T\right)$$

where *R_t_* denotes the resistance at *t* °C; *R_i_* is the resistance at *i* °C, and *α_T_* is the sensitivity (1/°C) [14].

Equation (1) can be rewritten as:

(2) $$\alpha_{T}=\frac{R_{t}-R_{i}}{R_{i}\;(\Delta T)}$$

## 3. Fabrication of Flexible Micro Temperature Sensor

The frame of the LED is a very important gateway for heat conduction for the LED chip, so a medium is installed between the frame of the LED and the LED chip to increase thermal resistance. In this investigation, micro temperature sensors were fabricated on a stainless steel foil substrate (SS-304 with 30 μm thickness), and aluminum nitride (AlN) was applied as an insulation layer because of its excellent insulation and high thermal conductivity properties.

**Figure 3.**
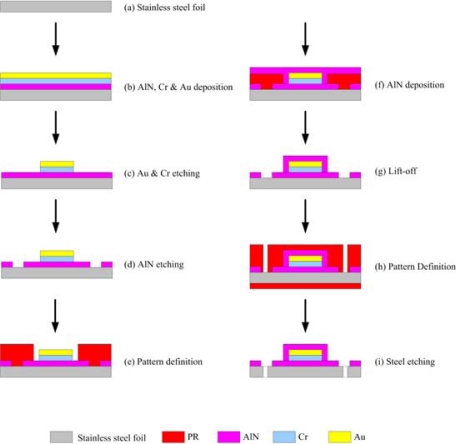
Fabrication of flexible micro temperature sensor.

The total line-length of the sensing area of the micro temperature sensor was 1,200 μm, and its planar dimensions were 230 × 90 μm^2^. Figure 3 presents the fabrication of a flexible micro temperature sensor using the following steps: first, sulfuric acid and hydrogen peroxide are employed to clean the stainless steel foil; the photoresist is then spun and lithography performed to define the outline of the micro temperature sensors. Then, aluminum nitride (AlN) is sputtered as a bottom insulation layer, and the lift-off method is used to remove the photoresist. An E-beam evaporator is then applied to evaporate chromium (Cr) as an adhesive layer between AlN and gold, and evaporated gold (Au) is used to form micro temperature sensors by wet etching. Finally, another aluminum nitride (AlN) with a thickness of 2,500 Å is sputtered as a top insulation layer, and the micro temperature sensors are connected via an Al wire.

## 4. Results and Discussion

**Figure 4.**
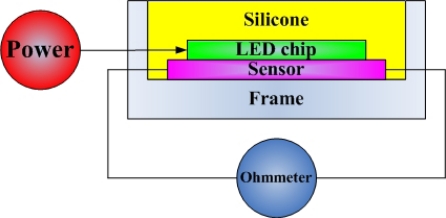
Illustration of micro temperature sensor in LED.

In Figure 4, the micro temperature sensor is set between a 1 W LED chip and a frame, and an input current with 350 mA is passed through the LED, causing it to glow. After the temperature has stabilized, the resistance of the micro temperature sensor is measured using an ohmmeter, and compared to the calibration curve of the micro temperature sensor, to determine the temperature; this result is compared the thermal resistance measurement. The temperature sensor requires a protective layer, so an insulation layer was deposited on the temperature, but the insulation layer was very thin, and the thermal gradient was small to ignore.

**Figure 5.**
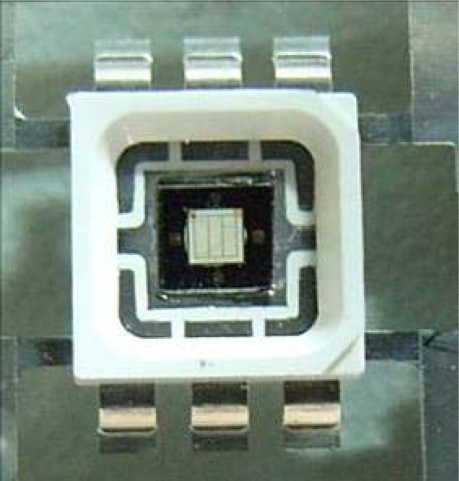
High-power LED with micro temperature sensor.

**Figure 6.**
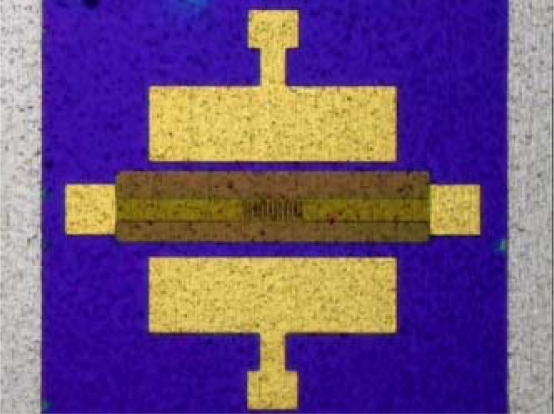
Optical microscopic photograph of micro temperature sensor.

**Figure 7.**
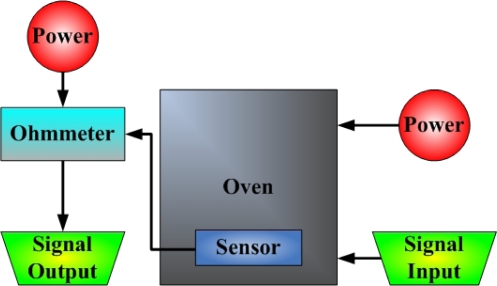
Calibration system of micro temperature sensor.

**Figure 8.**
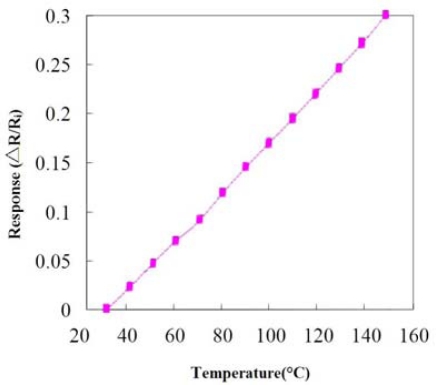
Calibration curve of the micro temperature sensor.

Figures 5 and 6 display an LED with a micro temperature sensor and a photograph of the micro temperature sensor, respectively. Figure 7 depicts the calibration system of a micro temperature sensor. The micro temperature sensor is placed in an oven, and the resistance of the micro temperature sensor is measured using an ohmmeter. Figure 8 plots the two calibration curves of the micro temperature sensor; the result demonstrates that temperature is almost linearly related to resistance and the sensitivity was 0.059 ± 0.004 Ω/°C, temperature sensor accuracy was defined based on the temperature in the accuracy range (0.5 °C) of programmable temperature chamber.

The junction temperature of the LED can be determined from the voltage based on the theory of thermal resistance measurement. A CREE® EZ1000 chip and input currents of 100 mA and 350 mA were used. The data obtained by thermal resistance measurement differ from those obtained using a micro temperature sensor, as shown in Table 1. The temperature obtained using the micro temperature sensor was 5.18 °C lower than that obtained by thermal resistance measurement at 100 mA, and 6.93 C lower at 350 mA. The difference increased with temperature.

## 5. Conclusions

In this work, a flexible micro temperature sensor that could be set between an LED chip and frame was developed, and 30 μm-thick stainless steel foil was used to conduct heat between the LED chip and the frame. Flexible micro temperature sensors were fabricated using micro-electro-mechanical-systems (MEMS) fabrication technique. The sensors have the advantages of: (1) small size, (2) high sensitivity, (3) flexible but precise measurement positions, and (4) mass production. A future study will adopt flexible micro temperature sensors to evaluate the operating parameters and thus improve the design and performance of LEDs.

## Figures and Tables

**Table 1.** Difference between measurements made using thermal resistance measurement technology and micro temperature sensor.

**Operating current**	**Thermal resistance measurement**	**Micro temperature sensor**	

	**Temperature (°C)**	**Resistance (Ω)**	**Temperature (°C)**
100 mA	52.3	23.82	47.12
350 mA	126.9	27.91	119.97
